# Residual hip dysplasia in children: osseous and cartilaginous acetabular angles to guide further treatment—a pilot study

**DOI:** 10.1186/s13018-019-1441-1

**Published:** 2019-11-21

**Authors:** Sophie Rosa Merckaert, Katarzyna Pierzchala, Aline Bregou, Pierre-Yves Zambelli

**Affiliations:** 1 Department of Women and Child’s Care, Unit of Pediatric Orthopedics, Centre Hospitalier Universitaire Vaudois, CHUV, Lausanne, Switzerland; 2 Center of Biomedical Imagery (CIBM), EPFL, Lausanne, Switzerland

**Keywords:** Residual hip dysplasia, MRI, Acetabuloplasty, Hilgenreiner’s angle

## Abstract

**Purpose:**

In case of residual hip dysplasia (RHD) in children, pelvic radiographs are sometimes insufficient to precisely evaluate the entire coverage of the femoral head, when trying to decide on the need for further reconstructive procedures.

**Methods:**

This study retrospectively compares the bony and the cartilaginous acetabular angle of Hilgenreiner (HTE) of 60 paediatric hips on pelvic MRI separated in two groups. Group 1 included 31 hips with RHD defined by a bony HTE > 20°. Group 2 included 27 hips with a HTE < 20°. They were compared by introducing a new ratio calculated from the square of cartilaginous HTE above the bony HTE on frontal MRI. The normal upper limit for this acetabular angle ratio was extrapolated from the published normal values of cartilaginous HTE and bony HTE in children.

**Results:**

The acetabular angle ratio was statistically significantly increased in the hips with RHD with a mean value of 7.1 ± 4.7 compared to the hips in the control group presenting a mean value of 2.1 ± 1.9 (*p* < 0.00001).

**Conclusions:**

This newly introduced ratio seems to be a helpful tool to orientate the further treatment in children presenting borderline RHD.

## Introduction

Developmental dysplasia of the hip (DDH) is defined as insufficient acetabular coverage of the femoral head [1]. It is one of the most frequent encountered congenital musculo-skeletal disorders among children [2]. Treatments of this paediatric disease range from conservative closed reduction to open surgical reduction [3–5]. Despite improvements in early detection and management [4, 6, 7], residual hip dysplasia (RHD) occurs in 3.5–17% of cases [6–8] and is a recognised risk factor for secondary osteoarthritis [7, 9–12].

Hilgenreiner’s angle (HTE) is routinely used for follow up on pelvic X-ray to assess the bony acetabular coverage [13, 14] and guide surgeons in their decision if further surgical correction is necessary. Its normal value at birth is below 30°, reducing rapidly in the child’s first 4 years towards 15 ± 5.5°, and staying stable until full hip ossification at maturity [13, 15–19]. RHD is defined as a HTE superior to 20° after the age of two [19, 20]. Despite those knowledge, there is still no consensus which degree of RHD will benefit from surgical correction after conservative treatment, especially for borderline RHD [21].

While plain radiographs evaluate the bony anatomy, they are insufficient to evaluate the cartilage and labrum, both of which contribute to the global coverage of the femoral head [22]. The cartilaginous part of the acetabulum seems to be an early and reliable predictor of acetabular development [20], as it’s fully formed at birth and, theoretically, it is supposed to represent the bony margins of the acetabulum at adulthood after full ossification [22–24].

An additional clinical tool for decision-making about the need for an acetabuloplasty in borderline RHD would be useful in daily paediatric orthopaedics’ practice. The purpose of the study was to evaluate a new measurement that we have termed the acetabular angle ratio (AAR). This is measured on pelvic MRI scans considering the bony and cartilaginous part of the acetabulum in order to help us in the decision process.

## Materials and methods

The present study was approved by the Human Research Ethics for analysis and subsequent publication of the identified data.

We retrospectively identified all children who had attended our orthopaedic centre for follow-up of DDH between 1997 and 2013. Inclusion criteria were conservatively treated children who had a pelvic MRI during follow-up aged between 1 and 8 years. Children with bilateral irreducible hip dislocation, previous surgery with acetabuloplasties and patient presenting hip dysplasia in association with cerebral palsy were excluded. Twenty children were eligible for the study (40 hips). RHD was defined by a bony angle of Hilgenreiner (O-HTE) > 20° [20]. In order to dispose about a control group, we included ten patients (20 hips) who had undergone a pelvic MRI for other purpose than DDH, which is not subject of the present study.

The 60 hips were divided in two groups. In group 1, we included 31 hips with RHD (O-HTE > 20°) from the study group; two hips were excluded because of irreducible hip dislocation. Group 2 included the 20 hips from the control group as well as the remaining seven hips with an O-HTE < 20° from the study group.

**Fig. 1:**
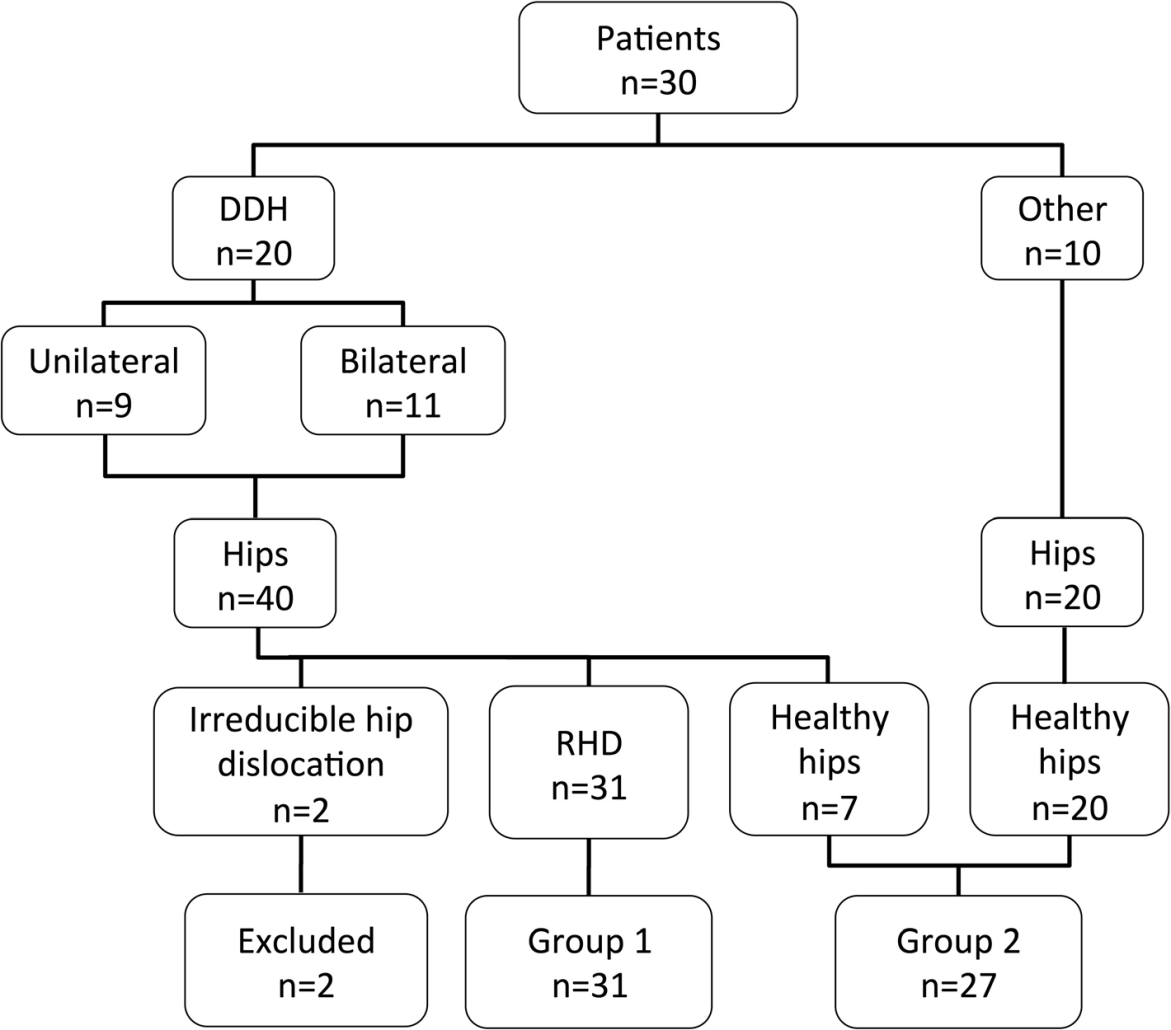
Flowchart, RHD (residual hip dysplasia)

The final distribution of the 60 hips can be seen in Fig. 1.

**Fig. 2:**
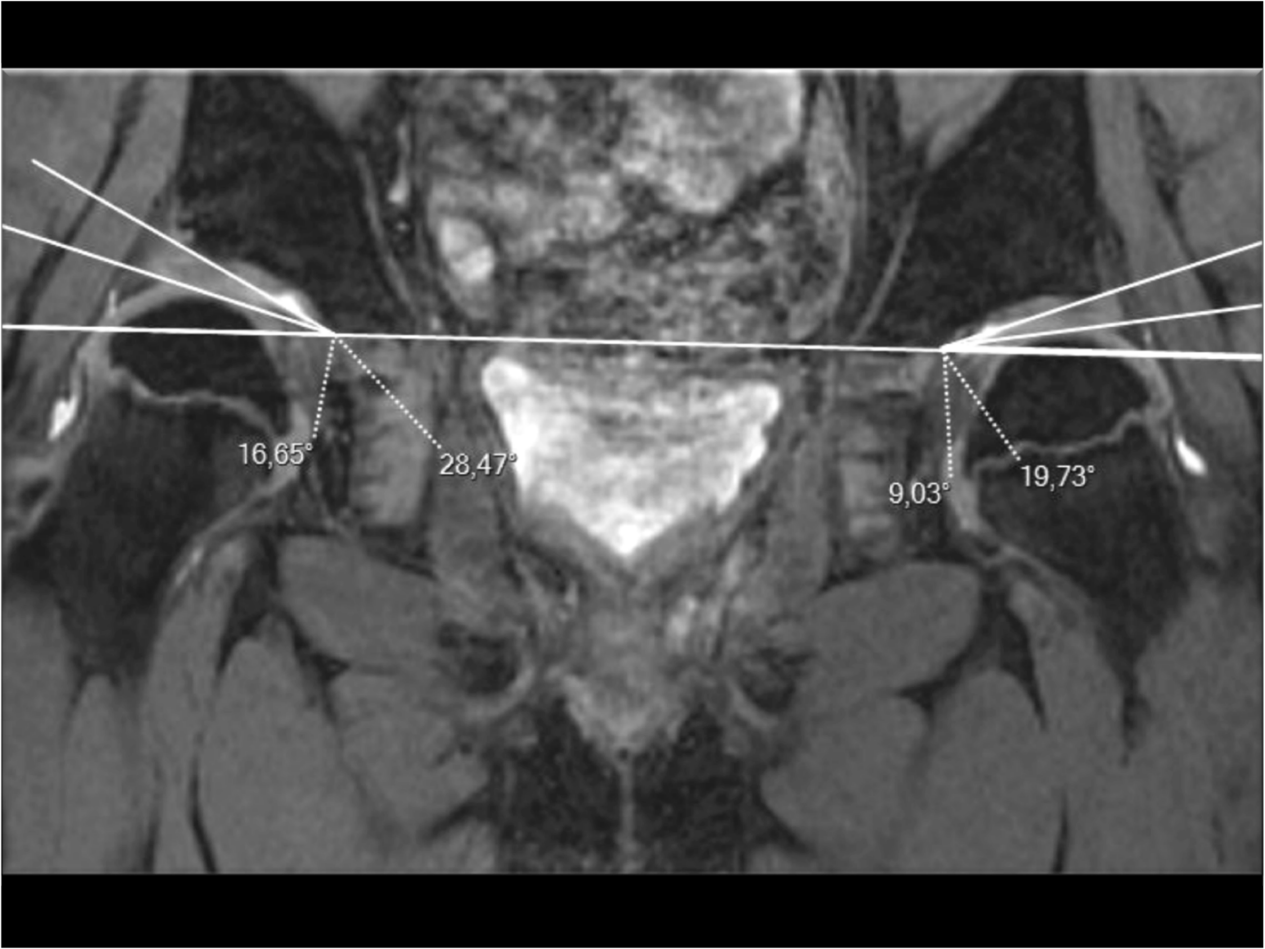
Measurements of Hilgenreiner’s angle on frontal MRI

For each hip, acetabular coverage of the femoral head was determined by the measurements of bony (O-HTE) and cartilaginous (C-HTE) angles of Hilgenreiner obtained from MRI double-echo steady state (DESS) or T_2_-weighted coronal scans of the pelvis. One orthopaedic surgeon using OsiriX imaging software performed all measurements. In order to be as close as possible to the centre of the hip joint, measurements of the acetabular angles were done on the frontal slice closest to the femoral epiphysis centres with the method described by Tönnis [15] (Fig. 2). All MRI were performed at Lausanne University Hospital’s Radiological Unit, on either Siemens Trio 3 T or Philips Trio 3 T MRI scanners, as well as Philips Avea 3 T and archived and viewed on PACS.

To get the overall head coverage, we reported the cartilaginous to the bony angle, using the ratio: (C-HTE)^2^/O-HTE. As C-HTE is always numerically inferior to O-HTE, we used the square of C-HTE, to obtain numerical values superior to one, in sake of clarification of the results without multiple decimal values.

From the published normal values of C-HTE and O-HTE in children [20], we extrapolated the normal limit value of this AAR.

The C-HTE in pre-school children tends to be from below 10° and the O-HTE angle is around 15 ± 5.5° [15, 20, 25, 26]. This set the limit of the ratio between a normal hip and a dysplastic hip at 5.

$$ \left[{\left(\mathrm{C}-\mathrm{HTE}\right)}^2/\mathrm{O}-\mathrm{HTE}={(10)}^2/(20)=5\right] $$

We hypothesised that hips presenting an AAR from above five are considered as pathological. Those hips would need further correction surgery as not only the bony part but also the cartilaginous part is insufficient

Statistical analysis was performed using OriginPro 8.5 software. Two-way unpaired Student *t* test was used for O-HTE, C-HTE and AAR comparison between the hips presenting RHD and the healthy hips. A *p* value < 0.05 was considered as significant.

## Results

Mean age in the 20 children followed up for DDH was 50 months (min 18, max 92) and 68 months (min 37, max 98) in the control group.

The demographic of those two groups is seen in Table 1.

**Table 1:** Demographics of the two study groups

Parameters	Study group	Control group
Number of patients	20 (40 hips)	10 (20 hips)
Age (months)	50 ± 18.2 (18–92)	68 ± 23 (37–98)
Gender (male)	3	7
Bilateral RHD	11 children	
Right hip RHD	4 children	
Left hip RHD	5 children	

Age is expressed as mean ± SD and range in parentheses, *RHD* residual hip dysplasia

RHD (O-HTE > 20°) was seen bilaterally in 11 children, in five children on the left side and in in four children on the right side. We recorded only three boys in our study group. Two hips were excluded because of irreducible high dislocation.

Mean O-HTE and C-HTE angles in hips with RHD (group 1) were 26.5 ± 5.2° and 13.4 ± 5.5°, versus 17.2 ± 3.6° and 5.3 ± 2.6° in group 2 respectively.

Sixty-five percent in group 1 had a C-HTE > 10° (20 hips). There was a statistically significant difference (*p* < 0.05) regarding C-HTE and O-HTE between group 1 and 2.

The calculated AAR presented a statistically significant difference (*p* < 0.00001) between group 1 and group 2, with a mean AAR value of 7.1 ± 4.7 in group 1 versus 2.1 ± 1.9 in group 2. The summary of our results is shown in Table 2.

**Table 2:** Comparative radiological measurement of acetabular angles

Group 1	O-HTE	C-HTE	AAR
1 RHD	26.5 ± 5.2	13.4 ± 5.5	7.1 ± 4.7
2 Healthy hips	17.2 ± 3.6	5.4 ± 2.8	2.1 ± 2.9
*p* value	< 0.00001	< 0.00001	< 0.00001

*O*-*HTE* bony acetabular angle, *C*-*HTE* cartilaginous acetabular angle, *AAR* acetabular angle ratio. Angles are given in degres and expressed as means ± SD

**Fig. 3:**
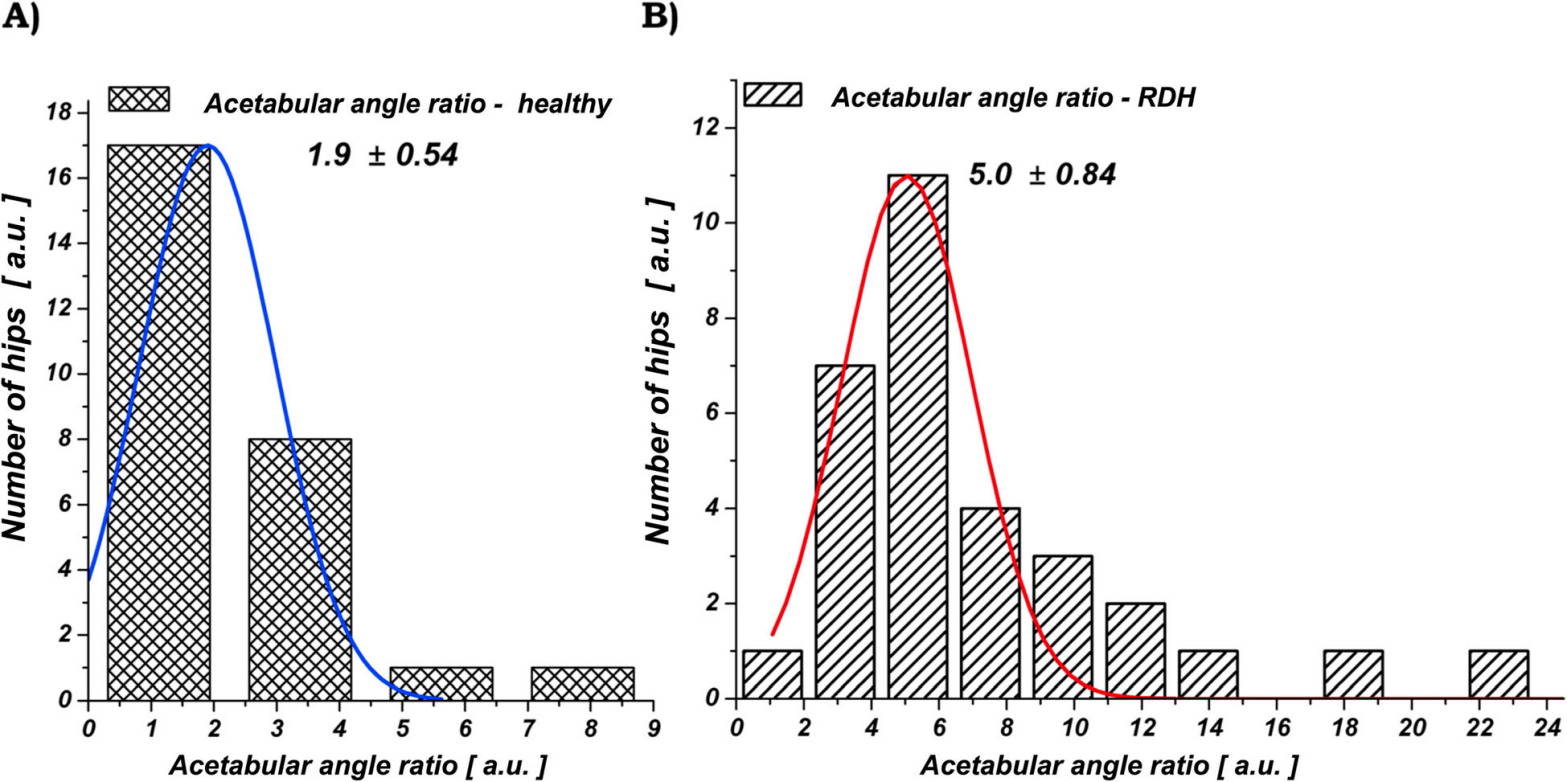
Distribution of the AAR Values for **a** Healthy hips (group 2) and **b** residual hip dysplasia (group 1)

The normal distribution of the AAR values was 1.9 ± 0.54 for normal hips in group 2 and 5.0 ± 0.84 for hips with RHD (group 1) (Fig. 3). The mean AAR values for hips presenting RHD increased by factor of 2.6 in comparison to healthy hip

## Discussion

The correct management of children presenting RHD after conservative treatment of DDH in some selected cases still represents a challenge [21].

Frontal plain pelvis radiographs are currently used to diagnose and assess RHD in children over 6 months of age and are almost a standard protocol in all paediatric centers across the world [27]. Hilgenreiner’s angle on pelvic x-rays seems to be the most used parameter for surgeons to decide if secondary surgery is needed [14] with a good inter- and intra-observer reliability [28], but as we know pelvic x-ray alone fails to give us any information about the fibro-cartilaginous parts of the acetabulum and may underestimate the residual growth potential of the acetabulum, leading in some cases to overtreatment [23]. On the other hand, some authors could show a significant variability between observers, as well as between time periods for a single observer, in the measurement of HTE and concluded that it is difficult to create clinical pathways to treat these patients and determine the impact of a certain treatment method over time [27].

It is widely accepted that a HTE greater than 20° after the age of four is defined as RHD [29], but we do not dispose of any precise radiological tool to help clinicians to decide whether a correction surgery of the acetabulum in those specific borderline cases of RDH is necessary or not [14, 30, 31]. Although we know that results of surgery for RHD are better in younger patients, there are still controversies over the indication for acetabular osteotomy in borderline cases of RHD [32, 33].

In our study, we set the upper age limit at eight, because acetabuloplasty performed after this age has been shown to lead to poor outcome and often other surgical procedures are needed, which is beyond the scope of this study [33]. Moreover, it is more difficult to draw Hilgenreiner’s line as ossification of the tri-radiate cartilage has already started [34].

The current definition for RHD relies on old concepts and ideas on the basis of pelvic radiographs that reflect only parts of the anatomic reality and are not treatment oriented.

Therefore, different studies have been published about the utility of MRI to predict further growth of the acetabulum in borderline RHD in order to help surgeons to decide if acetabuloplasty is needed, but no one could set a definite and objective value when to proceed to a corrective acetabuloplasty.

Bos et al. compared classic measurements on pelvic plain radiographs and MRI with bony and cartilaginous acetabular landmarks. MRI was found to be superior to radiographs by providing measurements of cartilaginous acetabulum and to arthrograms by distinguishing between labrum and limbus. They concluded that the highest risk of RHD exists when there is a lack of bony and cartilaginous coverage and recommended surgery in those cases. In case of insufficient bony coverage and sufficient cartilaginous coverage, they recommended close monitoring but they did not mention a clear cut-off value [35]. A similar study has been published by Douira-Khomsi et al. The authors concluded that MRI allows differentiation between bony and cartilaginous components allowing a more accurate selection of patients for pelvic osteotomy; unfortunately, no objective quantification of the acetabular coverage is given in this study [36].

Takeuchi et al. attempted to predict the future osseous acetabular development on the basis of cartilaginous acetabulum MRI evaluation in patients at 2 years of age. The authors measured the bony and cartilaginous HTE angles on MRI of 51 hips that were suspected to have RHD. They found the cartilaginous HTE to have a better-predicted value than the bony HTE and set a cartilaginous HTE of 13° as a cut-off value between a poor and a good outcome. Only six of 12 hips with RHD were in accordance with those prediction, which constituted a major limitation of this study [37]. Wakabayashi et al. introduced the ‘High-Signal Intensity Areas’ on T2-weighted MRI images, as a predictor for acetabular growth and as a decision-making tool for corrective osteotomy in borderline cases of RHD. However, borderline cases on which MRI is recommended are not well defined in this paper [38]. Huber et al. could define the normal values for bony and cartilaginous HTE on MRI from 115 hips in 73 children with a mean age of 7.3 years and showed that the cartilaginous HTE remains constant during growth. As they only included normal hips, further longitudinal studies are necessary studying hips with DDH and RHD to see if, and up to what age, a remodelling potential of the cartilaginous coverage exists [23].

Despite this knowledge regarding the normal values of C-HTE, there is still no consensus about when to perform an acetabuloplasty when RHD is seen and treatment of RHD after conservative treatment of DDH still remains mainly based on the clinical experience and on personal considerations of the treating surgeon [20, 39, 40].

MRI has the advantage not only to be non-irradiating but also to differentiate well between bony, cartilaginous and fibrous tissues and has been proved to be a useful tool for assessing RHD [25, 36, 38, 41]. As the fibro-cartilaginous labrum is hypo-intense on both T1-weighted and T2-weighted images, this makes it possible to differentiate the shape of fibro-cartilaginous structures in both sequences [42].

Furthermore, the measurements of O-HTE on MRI scan correlate with the O-HTE on plain radiographs [20].

It is thus especially suitable for pelvic studies in children [23, 34, 43].

Different studies set the normal value for the C-HTE angle at less than or equal to 10° [20, 23].

In our study, the average C-HTE and O-HTE angles from the 27 healthy hips in group 2 corroborated with previous researches [15, 19, 20, 23, 24].

The newly used AAR ratio, (C-HTE)^2^/O-HTE, was extrapolated from the already known normal values for the C-HTE and O-HTE.

We observed that 65% (20 hips) of the hips in group 1 presented an AAR above 5, and 16% (five hips) were even above 10. The remaining 35% (11 hips) had an AAR below five. In comparison, 96% (26 of 27) of hips in group 2 had an AAR below 5. The only AAR above five was discovered to be a non-diagnosed DDH until to the time the child has had an MRI for other purpose.

In the present study’s orthopaedic centre, the follow-up strategy for a patient with DDH is a plain radiography at 6, 12, and 48 months. If RHD (O-HTE > 20°) is still seen on pelvic plain radiography at the 4-year follow-up, pelvic MRI is performed; O-HTE and C-HTE angles are measured. AAR is calculated to guide us for further treatment decisions. An AAR above five means that not only the bony acetabular coverage is insufficient but also the cartilaginous part and therefore the hips has lower chance to normalise with growth, why we consider the need of surgical correction with an acetabuloplasty. Moreover, an AAR from below five, even if RHD is seen on plain radiographs, is thought to be a sufficient cartilaginous coverage with an O-HTE that has great chances to correct with growth and we therefore renounce to perform an acetabuloplasty.

The statistically significant difference in the AAR values between hips presenting RHD and the other hips in the control group could make the AAR a valuable decision-making tool in daily orthopaedic practice.

A disadvantage of the applied method is the procedure’s duration of the MRI because reliable images almost always require sedation or general anaesthesia of children at this young age [23, 34, 43].

The small number of patients and lack of intra- and inter-observer reliability in our study, even if previous studies agree that the measurement of Hilgenreiner’s angle show good inter- and intra-observer reliability [28], also represents a weak point.

By using a standard radiological technique, this method may allow to apply a common decision algorithm to every child presenting a residual hip dysplasia by the age of four. Another advantage of this method is that we avoid measurement errors of the HTE on plain radiographs due to pelvic tilt or rotation [44]. With an AAR from below five, we do not consider the need for acetabular correction surgery, whereas with an AAR above five we recommend surgical correction.

Further studies with comparison of the results from children presenting RDH that had no surgery with an AAR above 5 are needed to assess the potential role and validity of the AAR.

## Conclusion

The AAR (C-HTE^2^/O-HTE) could be a useful tool to guide us in the decision process for further surgical treatment in hips presenting borderline RHD after an initial conservative treatment of DDH.

## Data Availability

The datasets used and/or analysed during the current study are available from the corresponding author on reasonable request.

## Abbreviations

C-HTE: Cartilaginous Hilgenreiner’s angle
DDH: Developmental dysplasia of the hip
O-HTE: Osseous Hilgenreiner’s angle
RHD: Residual hip dysplasia
